# Cell-cycle synchronization reverses Taxol resistance of human ovarian cancer cell lines

**DOI:** 10.1186/1475-2867-13-77

**Published:** 2013-07-30

**Authors:** Xueqing Wang, Lingya Pan, Ning Mao, Lifang Sun, Xiangjuan Qin, Jie Yin

**Affiliations:** 1 Department of Obstetrics and Gynecology, Beijing Jishuitan Hospital, The Fourth Teaching Hospital of Beijing Medical College, Beijing, China; 2 Department of Obstetrics and Gynecology, Peking Union Medical College Hospital, Chinese Academy of Medical Science and Peking Union Medical College, Peking, China; 3 Department of Cell Biology, Institute of Basic Medical Sciences, Academy of Military Medical Sciences, Beijing, China

**Keywords:** Thymidine, Cell cycle, Chemoresistance, M stage, Cell-cycle synchronization

## Abstract

**Background:**

Taxol is a powerful chemotherapy agent leading to mitotic arrest and cell death; however, its clinical efficacy has been hampered due to the development of drug resistance. Taxol specifically targets the cell cycle. Progress through mitosis (M stage) is an absolute requirement for drug-induced death because cell death is markedly reduced in cells blocked at the G_1_-S transition. The measured doubling time for ovarian cancer cells is about 27 h. As such, during treatment with Taxol most of the cells are not in the M stage of the cell cycle. Thus, the effect of cell-cycle synchronization was investigated in regard to reversing Taxol resistance in ovarian cancer cells.

**Methods:**

Giemsa-Wright staining was used for assessing the morphology of the cells. The doubling time of the cells was calculated using formula as follows: Td = In2/slope. The resistant index and cell cycle were measured via MTT assays and flow cytometry. Thymidine was used to induce cell-cycle synchronization, and cell apoptosis rates following exposure to Taxol were measured using a flow cytometer.

**Results:**

The growth doubling time of two Taxol-resistant cell lines were longer than that of Taxol-sensitive cells. Apoptotic rates in Taxol-sensitive and -resistant cell lines after synchronization and exposure to Taxol were all higher compared to unsynchronized controls (p <0.05).

**Conclusions:**

Synchronization of the cell-cycle resulted in an increased effectiveness of Taxol toward ovarian cancer cell lines. We speculated that formation of drug resistance toward Taxol in ovarian cancer could be partly attributed to the longer doubling time of these cells.

## Introduction

Ovarian cancer is the third leading cause of death and has the highest mortality rate among the gynecologic malignancies. Because of the effectiveness of Taxol on uncontrolled ovarian cancer, Taxol has quickly become the first-line chemotherapy treatment [1-3]. Taxol has high cytotoxic action on many types of cell lines in vitro, especially ovarian, breast, and lung [4-6]. Although combination chemotherapy, such as Taxol and cisplatin, has improved the prognosis for the initial treatment of ovarian cancer, the 5-year survival rate of advanced-stage ovarian cancer is still between 15-20%, due to the emergence of a broad resistance pattern that is either intrinsic to the tumor or acquired after chemotherapy [7-11]. Acquired resistance to taxol was investigated in the current study.

Taxol was first isolated from the bark of the western yew and has been shown to have cytotoxic activity against a wide range of neoplasms. Taxol is an anti-mitotic agent that binds to microtubules and stabilizes them against depolymerization; therefore, Taxol inhibits cell replication by disrupting normal mitotic spindle formation and arresting cell growth in the M phase of the cell cycle [12-14]. Reversal of drug resistance in cancer chemotherapy is a complex phenomenon involving diverse molecular mechanisms. Currently, research on drug resistance involving Taxol has been associated with induction of the multidrug resistance (MDR) phenotype, overproduction of p-glycoprotein, mutation of tubulin sites, and abnormal expression of bcl-2 [15-19]. Indeed, such research concerning Taxol resistance emphasizes alterations during the cell cycle.

Taxol induces apoptosis by blocking cells in the G2/M phase of the cell cycle. Although several studies have suggested a correlation between drug resistance and the cell cycle, the exact mechanisms have not been fully investigated. As such, drug resistance at the molecular level still requires further investigation [20,21]. Normal cells proliferate through the G1, S, G2, M, and G1 stages via serial, strictly monitored mechanisms. Cells with abnormal cell-cycle progression typically die after undergoing apoptosis. The nature of cancer is related to alterations in the mechanisms influencing the cell cycle. The mechanism of action of many kinds of anti-tumor drugs on cancer cells is attributed to the disturbance of cell-cycle control [22-24]. Taxol (also known by its generic name paclitaxel) is known to invoke a mitotic checkpoint; however, the full mechanisms of action remain incompletely characterized. Cells that are relatively resistant to these drugs block mitosis, whereas cells sensitive only transiently block mitosis before undergoing nuclear fragmentation and death. Passage through mitosis is an absolute requirement for Taxol-induced death because death is markedly reduced in cells blocked at G_1_-S and G_2_[25,26]. The cell cycle reflects the station of a group of cells rather than a single cell. While growing in the same medium, all cells are not at the same stage and concordance is absent. This greatly reduces the efficacy of Taxol. The replication time of some ovarian cancer cells is approximately 27 h and resistant cell lines even more longer. During the whole cells cycle, most cells were in G0/G1 or S stage [27]. Thus, a disparity exists between the longer doubling time of cancer cells and the shorter window of action in which Taxol functions, as such most cells do not occupy the M stage during the short window of Taxol action. Synchronization of the cell cycle via thymidine results in arrest of most cells at the S stage because DNA synthesis is not completed and the cells cannot proliferate to the next stage (M phase). After the medium containing thymidine is changed with common medium, most cells gradually enter the M phase at approximately the same time. The effect of cell-cycle synchronization via thymidine on reversing Taxol resistance in epithelial ovarian cancer cell lines was investigated in this current study.

## Materials and methods

### Drug and reagents

Taxol was obtained from Bristol-Myers Squibb Co. (Princeton, NJ, USA) and stored at a concentration of 10 nM diluted in DMEM at room temperature. High-glucose medium (HG-MEM) was purchased from Gibco BRL (Grand Island, NY, USA). MTT and DMSO were purchased from Sigma-Aldrich (St. Louis, MO, USA). RNase A was purchased from Beijing NuPu Biological Company (Beijing, China). A cell apoptosis kit was purchased from Beijing BaoSai Technological Corporation (Beijing, China). Thymidine was purchased from Merck & Co (Whitehouse Station, NJ, USA).

### Cell lines and cell culture

The SKOV3 cell line was obtained from the Biological Cell Institute of Chinese Peking Union Medical College (Chinese Academy of Medical Science). The Taxol-resistant cell line, SK-TJ2500, was induced from SKOV3 cells by intermittent exposure to 2.5 μM Taxol. While growing to the logarithmic phase, cells were given repeat stimulation with Taxol for approximately 1 h over a period of 16 months. A2780 and its Taxol-resistant cell line, TA2780, were obtained from the Oncologic Institution of Guang Xi Medical College. All the cells were maintained in Dulbecco’s modified Eagle’s medium (DMEM) containing 10% fetal calf serum, 100 U/ml penicillin and streptomycin. Cells were kept at 37°C in a humidified atmosphere of 5% CO2 and 95% air. These cell lines grew in monolayers and were passaged when cultures were 70-80% confluent.

### Morphological observations

For light microscopy, exponentially growing cells were transfered to 30-mm dishes containing sterile glass slides and allowed to adhere in 5% CO2 at 37°C for several days. When the cells were 70-80% confluent, the slides were washed, fixed in methanol for 10 min and stained by the Wright-Giemsa method. (Philips, Eindhoven, The Netherlands).

### Resistant index (RI) and MTT assays

Cells were harvested from exponential phase and digested using 0.25% trypsin. Single-cell suspensions were prepared. Cells were counted using a hemocytomhemo cytometer and then dispersed within 96-well microtiter plates. Six duplicate wells were used for each determination. Plates were maintained at 37°C in a humidified atmosphere of 5% CO2 and 95% air. A 24-h preincubation time was allowed prior to addition of drugs. Taxol were added to each well in six grades. After incubation of 72 h, MTT (5 g/l) 20 ul was added to each well. Four hours later, 100 ul of DMSO was added and incubated for 10 min at room temperature. Absorbance at 540 nm on each well was measured using Immunoskan 340 (Labsystems, Vantaa, Finland). Control wells for absorbance readings contained cell-free medium. All experiments were performed at least three times. Resistance index (RI) equals the ratio of the inhibitory concentration 50% (IC50) values of resistant to sensitive cells.

### Doubling time assays

Cells were added to 24-well plates (5 × 10^3^ cells/well) and cultured at 37°C with 5% CO_2_. Three duplicate wells were used for each determination. Four cell counts for each replicate from each cell line were made every 24 h for 7 days. The data were subjected to liner regression analysis, in which the doubling time (Td) was calculated from the formula: Td = In2/slope.

### Cell-cycle analysis by flow cytometry

Monodispersed cells 1×10^6^ were harvested during the exponential growth phase.

The cells were washed with PBS, fixed in 70% ethanol, and stored at -20°C overnight. The fixed cells were washed twice in PBS, resuspended in PBS containing 200 μg RNase A (Sigma) and incubated at 37°C for 30 min. The samples were stained with 20 μg propidine iodide protected from light for 30 min and the distribution of cells in the cell cycle was analyzed using a flow cytometer. (BD Company, Franklin Lakes, NJ, USA). All experiments were performed at least three times.

### Cell-cycle synchronization

When cells growing in 6-well plates had grown to 60-70% confluence, 2 mmol/L thymidine was added to the medium. After 11-16 h of culture, the culture medium was replaced with fresh medium, and the cells were subsequently treated with thymidine for an additional 16 h (Harper JV 2005 UK). Then, the cells were separated into two groups. One group of cells was collected for cell-cycle analysis and the other group of cells continued culturing and Taxol (100 nmol/l) was added to the medium 8 h later and maintained for 4 h. Finally, the cells were collected 48 h after Taxol was withdrawn, and cell apoptosis rates were determined using a flow cytometer. All experiments were performed at least three times.

### Apoptosis assays

After trypsinization and centrifugation, the cells (between 5×10^5^ and 1×10^6^) were rinsed twice with PBS and the supernatant was discarded. The cells were then suspended in 0.2 ml of buffer. Annexin-V FITC (10 ul) and PI (5 ml) were added in the dark. The cell suspension was mixed slightly and maintained at room temperature for 15 min and then at 4°C for 30 min. Buffer (0.3 ml) was added to detect the cell apoptotic rate using a flow cytometer over a 1 h period (baosai Technological Corporation Beijing china). The same experiment was repeated at least three times.

### Statistical analysis

The experimental data are shown as the mean ± SD. Employing SPSS v.12.0 software (SPSS, Chicago, IL, USA), a *t*-test was used to assess pairwise differences between the treatment and control groups. A p-value of <0.05 was considered statistically significant.

## Results

### RI and group doubling times

**Figure 1:**
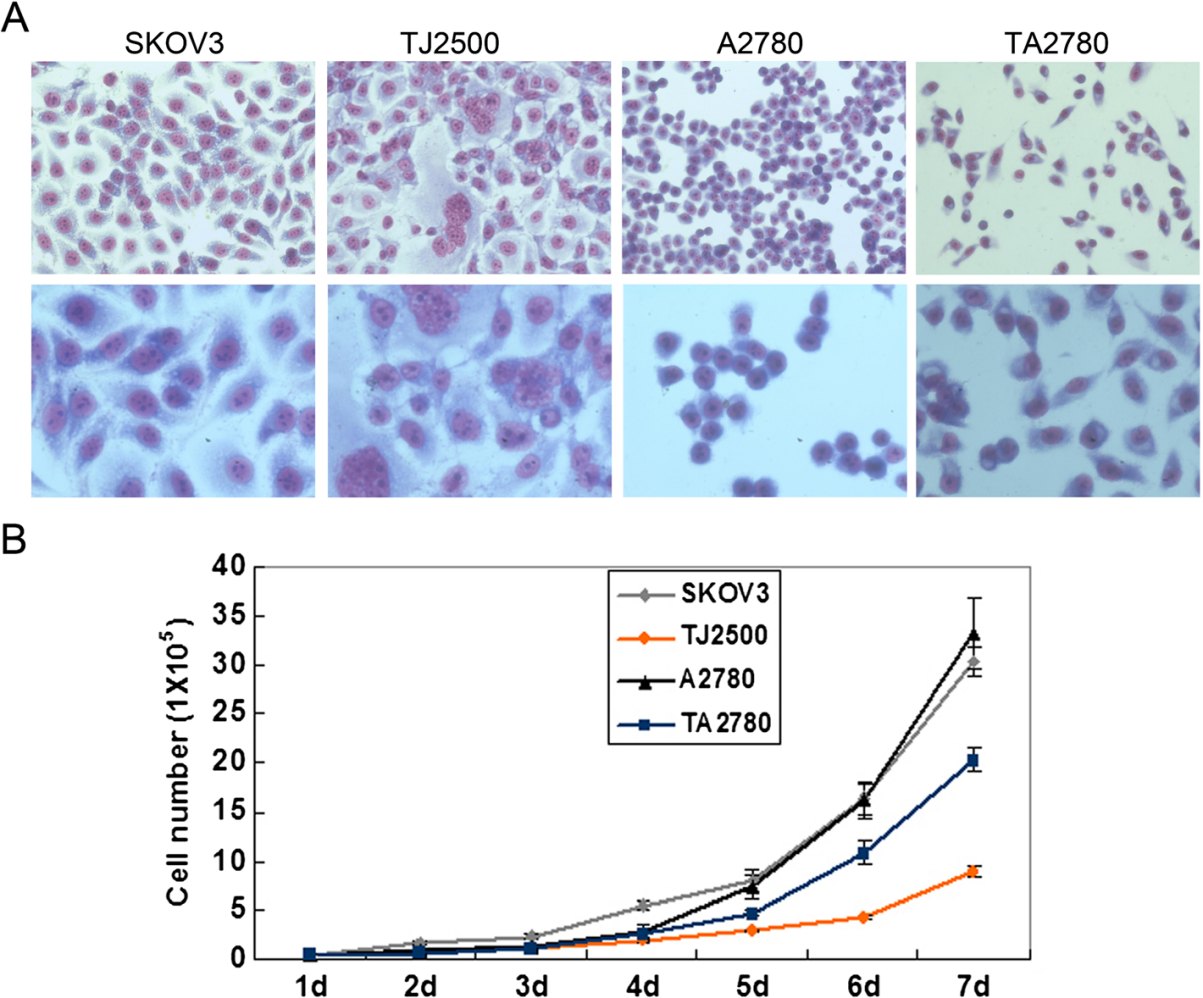
**Images and corresponding growth curves for the four cell lines.** Legend **A**: morphology of four cell lines by inverted microscope at original magnification × 20 and 40 respectively. Legend **B**: The cell growth curves of the human ovarian cancer cell lines. SKOV3, A2780 and their drug resistant sublines TJ2500 and TA2780. Four cell counts for each replicate from each cell line were made every 24 h. for 7 days. Cell counts using trypan blue exclusion to assess viable cells were used to determine Td for each cell line. Td was calculated as described in Materials and methods.

Table 1 shows the different RIs of the sensitive and resistant cell lines. The growth doubling time for the sensitive cell lines (SKOV3 and A2780) were about 27 h and that of resistant cell lines (TJ2500 and TA2780) were statistically longer and difference is significant. Growth curves for the four cell lines are shown in Figure 1.

**Table 1:** Resistant index and group doubling time of four kinds of cells

**Cell lines**	**Doubling time(hours)**	**Resistang index(RI)**
SKOV3	27.49 ± 4.21	1
TJ2500	37.61 ± 3.34**	62.35 ± 11.3
A2780	27.07 ± 8.58	1
TA2780	31.23 ± 6.624*	25 ± 6.5

Legend: **(p<0.01) compared with SKOV3, * (p<0.05) compared with A2780.

Analysis of sensitive and resistant cells stained with Giemsa (Figure 1) showed that aberrant nuclei among TJ2500 cells were more prevalent than among SKOV3 cells and that the appearance of the TA2780 cells was mostly fusiform compared to the round shape of A2780 cells. We speculated that these changes in morphology might be related to their sensitivity and resistance toward Taxol, as these biological properties likely reflect the growth and fission of cells.

### Cell-cycle analysis

**Figure 2:**
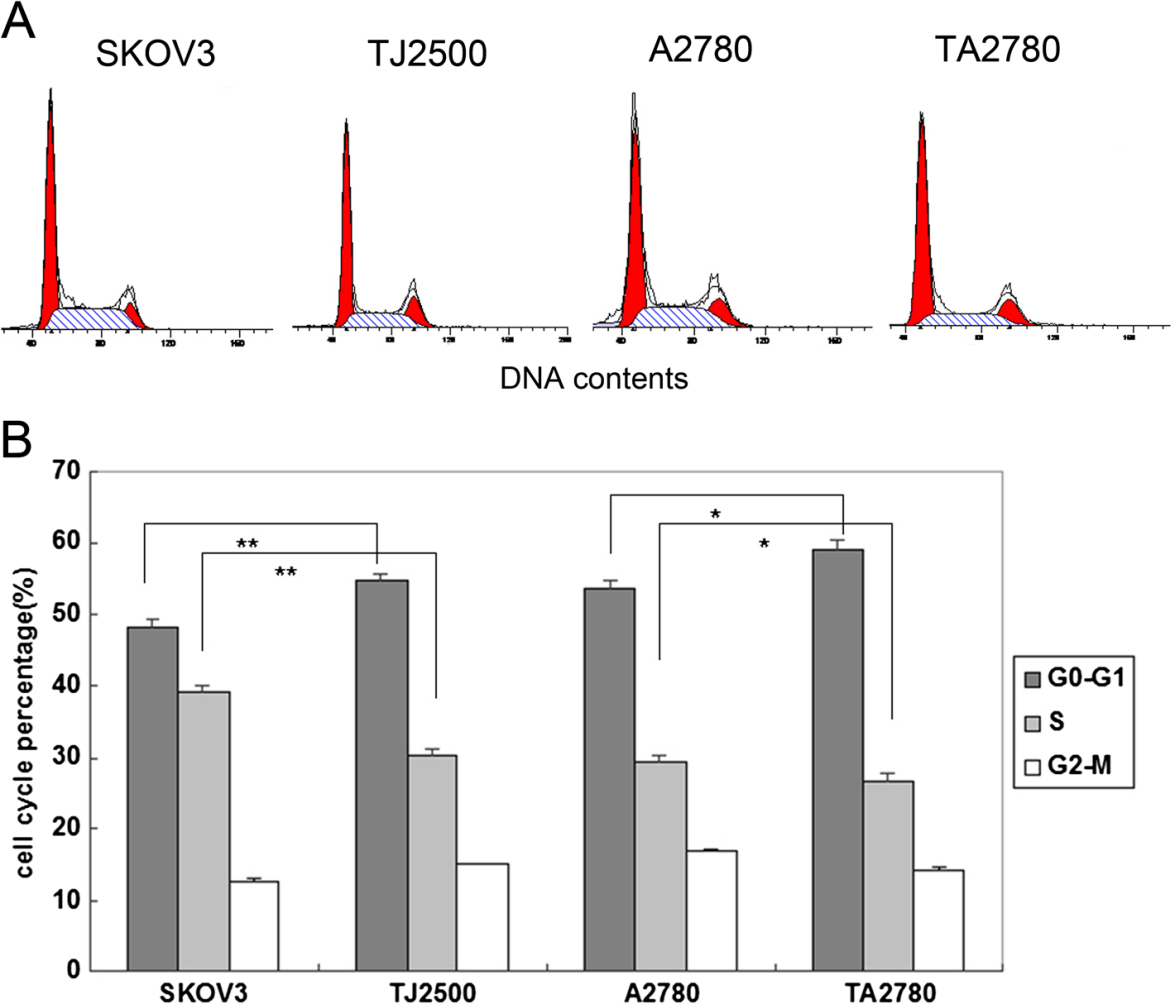
**Cell cycle analysis for the four cell lines.** Legend **A**: the results of cell-cycle analysis by flow cytometry the initial and last red peak represent G0-G1, G2-M stage respectively, middle is S stage. Legend **B**: The percentage of resistant cells in G0-G1 was increased and reduced in the S phase compared with sensitive cells. **(p<0.01) compared with SKOV3, * (p<0.05) compared with A2780.

Cell cycle analysis was carried out to further investigate the longer doubling times of the resistant cell lines compared to the sensitive cell lines. Results in Figure 2 show that the resistant cell lines have a significantly higher proportion of cells existing in the G0-G1 stage of the cell cycle and a significantly reduced number of cells in the S phase compared to the sensitive cell lines. However, the number of cells in the M stage was not changed significantly. The increased number of cells existing in the G0-G1 stage in the resistant cell lines may reduce the potency of Taxol.

### Cell-cycle synchronization with thymidine

**Figure 3:**
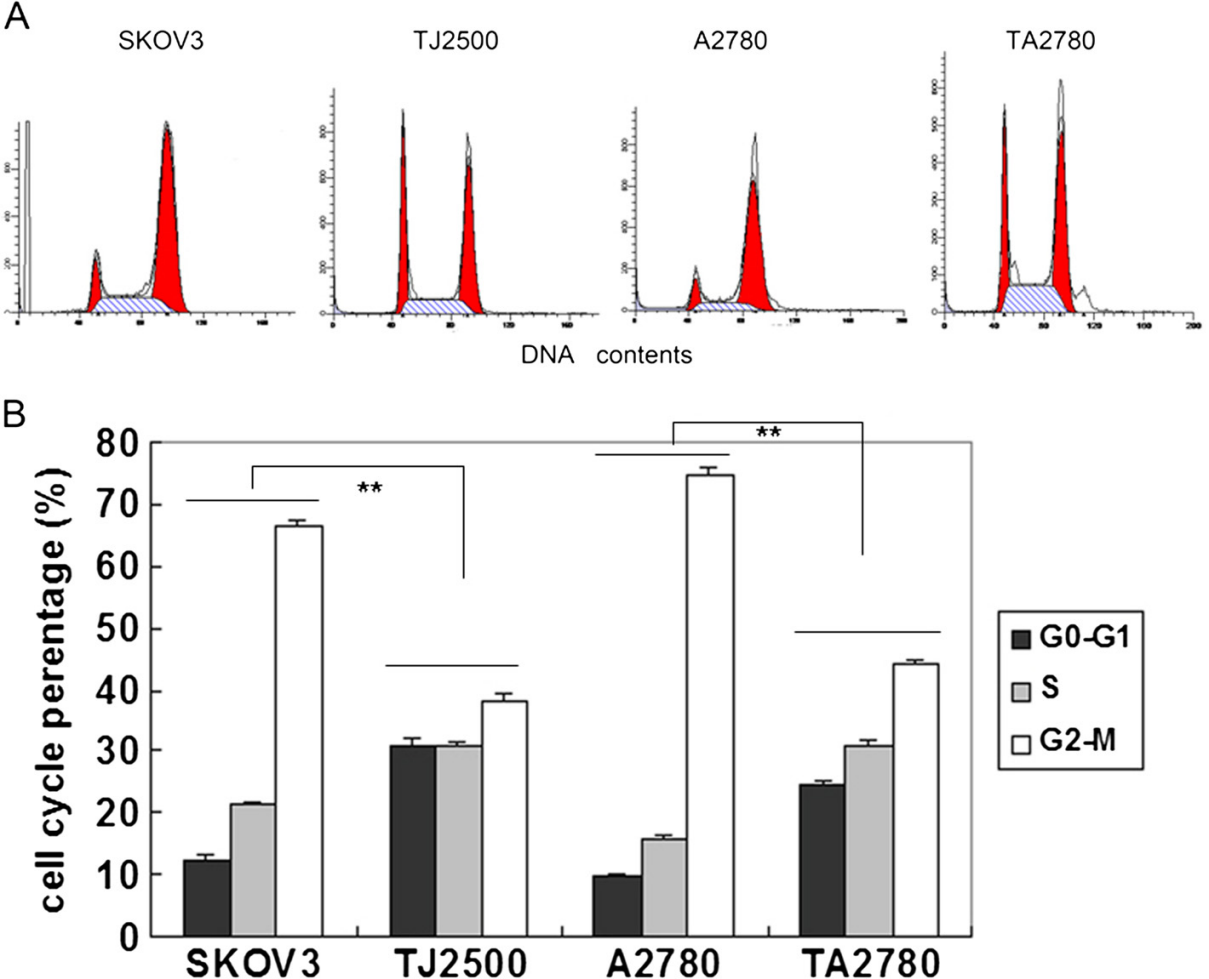
**The percentage of different phases of cell cycles synchronized with thymidine and changed with common medium 8 h later.** Legend **A**: the results of cell-cycle analysis by flow cytometry in cells synchronized with thymidine and changed with common medium 8 h later. the initial and last red peak represent G0-G1, G2-M stage respectively, middle is S stage. Legend **B**: percentage of cells in the M stage was greater than those cells without synchronization for all four cell lines. At the same time point after synchronization, significantly more sensitive cells were in the M stage compared to resistant cells, difference is significantly. ** (p<0.01).

In order to test the above assumption, we added thymidine to the medium, which prohibited the synthesis of DNA and hampered the progression of the cells into the next stage, resulting in most of the cells arresting in the S phase. The proportion of cells in the G2/M phase was subsequently reduced. Following the removal of the thymidine, most of the cells gradually entered the M phase several hours later. The time at which most sensitive cells (SKOV3) synchronized with thymidine entered the M phase was approximately 6-8 h; the Taxol-resistant cell line (TJ2500) required 8-10 h. In contrast to Figure 2, the results in Figure 3 show that with the addition of thymidine, more cells of the sensitive cell lines (SKOV3 and A2780) had entered the M phase compared to the resistant cells lines (TJ2500 and TA2780) when assessed at the same time point. This indicated that the speed at which the resistant cells proliferated was slower compared to the sensitive cell lines. We therefore choose 8 h as the time point to add Taxol and to assess apoptosis after synchronization.

### Cell apoptotic rates

**Figure 4:**
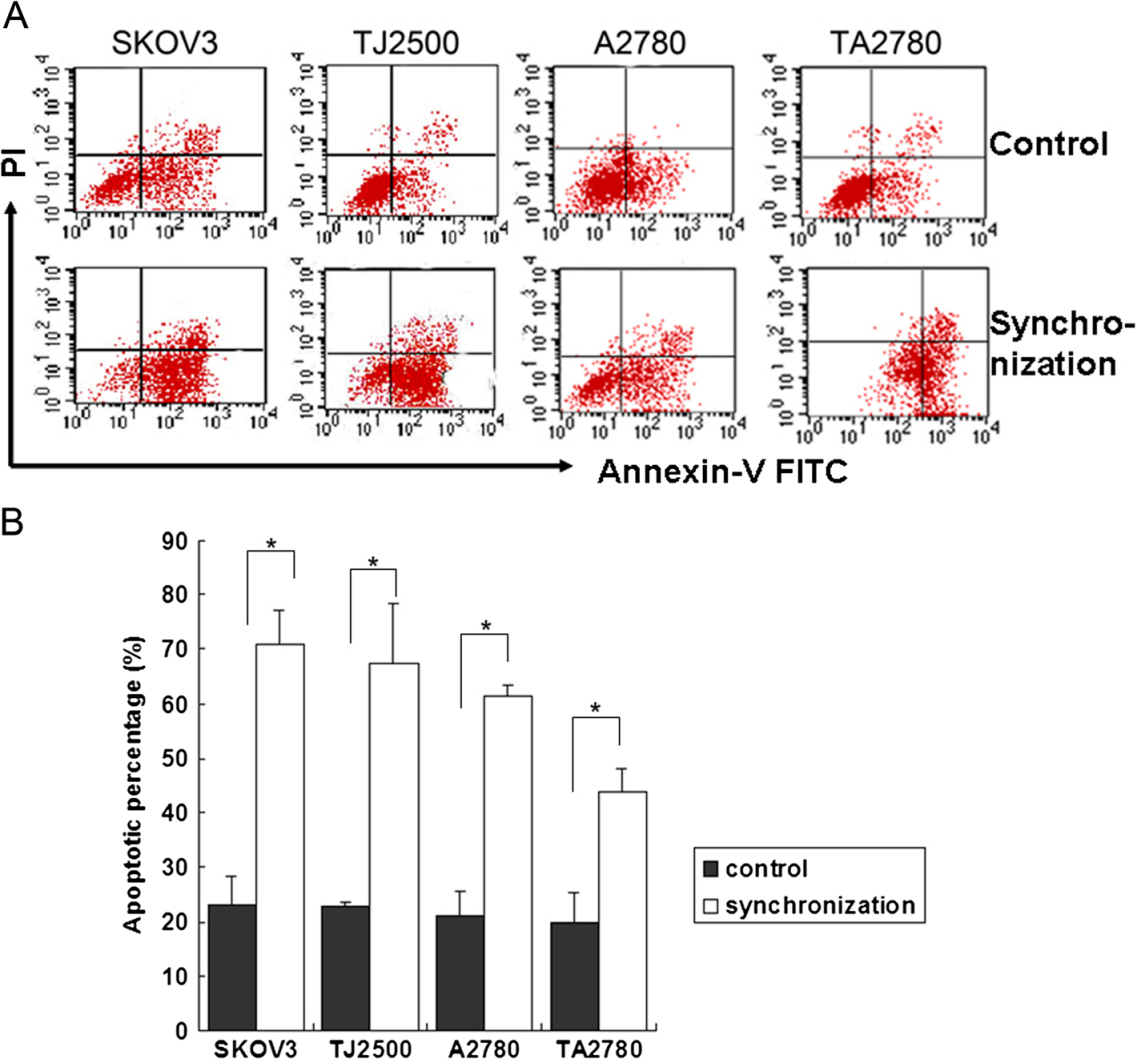
**Comparison of apoptotic rates before and after synchronization for the four cell lines.** Legend **A**: two Quadrant on the right of figure is apoptosis rate by flow cytometer. Legend **B**: Taxol was added at the time when most cells were in the M stage. The results showed that the apoptotic rates in Taxol-sensitive and -resistant cells after synchronization were higher than the unsynchronized controls (p<0.05), especially in sensitive cell lines. Results are an average of 3 independent experiments.

After synchronization with thymidine, cell lines (both sensitive and resistant) were incubated in common medium without thymidine for about 8 h and then stimulated with Taxol (100 nmol/L) for 4 h. The apoptosis rates of the cells were detected after withdrawed Taxol 48 h later. Independent trials were performed at least in triplicate. Figure 4 shows the significantly different apoptotic rates among the four cell lines compared with unsynchronized controls (p <0.05). Furthermore, apoptotic rates of the resistant cells after synchronization were lower than that of the sensitive cells, especially in regard to the TA2780 cell line.

## Discussion

Microtubules represent effective targets in cancer therapy. Taxol, a member of the Taxane family of anticancer drugs, stabilizes microtubules. At the molecular level, Taxol binds directly to microtubules, leading to a potent suppression of microtubule dynamics, increased microtubule stabilization, and interphase microtubule bundling; consequently, cells undergo robust mitotic arrest and subsequent apoptotic cell death. The mitotic checkpoint is thought to be invoked by these microtubule-disrupting drugs via mechanisms that monitor correct spindle formation and tension. In normal cells this checkpoint helps ensure that equal numbers of chromosomes are distributed to daughter cells and, thus, avoid mis-segregation and the potentially catastrophic consequences of aneuploidy [28,29]. The development of protocols using RNA interference to specifically silence gene expression has provided the ability to test the potential function of specific components of this checkpoint. Our prior research found that these drugs induced nuclear fragmentation mainly after cells had progressed into mitosis; conversely, the lethal effects of these drugs were significantly lessened when cells were prevented from progressing into mitosis [26]. Current models propose that the mitotic checkpoint proteins (BubR1, Bub1, Bub3, Mad1, and Mad2) sense a lack of tension or attachment between the kinetochore and microtubules of the mitotic spindle and transmit a “wait signal” to inhibit the anaphase-promoting complex [30,31]. Certain cancer lines evolved a less robust mitotic checkpoint, and such defects in the mitotic checkpoint may confer a growth advantage by enabling cells to tolerate chromosomal instability or aneuploidy or by allowing faster proliferation. In support of the latter, Shichiri et al. found few mutations of BubR1 or Bub1 in a series of surgically resected colorectal tumors, but the subset that showed low mRNA expression was associated with a significantly higher recurrence rate [30]. The results of Lee et al suggested that cell death after drug treatment with Taxol in SkBr3 cells was a consequence of entering mitosis. To formally exclude the possibility that cell death may result earlier from effects at other phases of the cell cycle, they synchronized SkBr3 cells in G1-early S phase and followed their progression through the cell cycle following release and drug treatment. The addition of Taxol 2 h after release did not have a discernable effect on progression into either the S or G2 phase compared with control cells. However, at a time when the control cells had largely completed mitosis and returned to the G1 phase, cells treated with Taxol showed substantial nuclear fragmentation and death [26]. Experiments have also suggested that the integrity of the mitotic checkpoint is an important determinant of sensitivity of cancer cells toward microtubule-disrupting chemotherapy and that resistant cell lines show a robust mitotic checkpoint, including strong expression of the BubR1 and Mad1. The drug resistance of these cells could also be reversed by silencing of BubR1 [26,32]. However, others have provided evidence that expression of BubR1 is reduced and the function of the spindle checkpoint is weakened in cell lines with Taxol resistance [33,34]. Drug resistance is often a multifactorial process, and although we cannot exclude the possibility that mechanisms distinct from the spindle checkpoint contribute to Taxol resistance, there must exist other factors affecting formation of drug resistance during cell cycle. Passage through mitosis is an absolute requirement for drug-induced death. It is tempting to speculate that the clinical response of certain tumors to microtubule-disrupting drugs may be at least partially reflected in the intrinsic sensitivity of cell lines derived from these tumors. For example, the efficacy of Taxol in treating patients with breast and ovarian cancer is well established, whereas results in treating sarcomas, colorectal, and renal cancer have been disappointing [35-37]. It is likely that these mitotic checkpoint proteins participate in different pathways to effect orderly mitotic progression; as such, their relationship with Taxol resistance requires further investigation.

The focus of this current study was on the formation of acquired drug resistance. The resistant cells lines in this experiment were obtained by intermittent exposure of sensitive cells to Taxol. The group doubling time of ovarian cancer lines (SKOV3 and A2780) were approximately 27 h, and that of resistant cells (TJ2500 and TA2780) was even longer. Figure 2 show cell cycle of four cells line which unsynchronizated. cells in G0-G1 and S stage occupied 80-90%, M-stage only 10-20%, especially resistant cells (TJ2500, TA2780) in G0-G1 stage much more than sensitive cells lines, difference is significantly. While growing in the same medium, cells are not all at the same stage of the cell cycle and concordance is absent, which results in a lower efficacy of Taxol, as its effectiveness is limited to a short time period while cells in M stage. Only over a treatment time of approximately 27 h could it be possible to conclude that most cells had passed through the M stage. We speculated that formation of drug resistance toward Taxol in ovarian cancer could be partly attributed to the longer doubling time of these cells.

We used cell-cycle synchronization to test the above assumption. After adding a high concentration of thymidine, most of the cells arrested at the S phase and could not progress to the next stage (M phase). After a fresh medium change, most of the cells gradually entered the M phase at nearly the same time, at this point Taxol was added to the medium. Figure 3 show cell cycle of four cells line which synchronizated. cells in G0-G1 and S stage particularly ruduced and M-stage much increased, especially sensitive cells(SKOV3,A2780) in M stage more than resistant cells lines. The apoptotic rates of cells treated with thymidine were significantly increased compared with the cells without thymidine, and this was not only observed in sensitive cells but also in resistant cells. Taxol-resistant cells may require a longer time of action when compared to sensitive cells in order to achieve optimal results; this is because the proliferation period or growth doubling time of the resistant cells was longer and most of these cells were in the non-M phases during the window of Taxol action.

Cell-cycle synchronization resulted in an increase in the number of cells passing through the M stage at a given time and reduced the toxicity of Taxol toward cells in the non-proliferative phase, improving its effectiveness and decreasing the chance of drug-resistant formation. This method may be an efficient approach to strengthen the effectiveness and reverse drug resistance related to Taxol.

However, apoptosis of resistant cells after synchronization was lower than that of sensitive cells, especially in regard to TA2780 cells. This observation partly implied that resistance formation towards Taxol is a complex phenomenon involving diverse molecular mechanisms, as opposed to a single factor. The observation that entry into mitosis seems to be a requirement for rapid killing after microtubule disruption in drug-sensitive cells has implications for the sequential application of these drugs and other forms of anticancer treatment. For example, initial use of Taxol may reduce the lethal effects of microtubule-disrupting drugs. Thus, it may be preferable to administer therapy inducing cell-cycle synchronization before paclitaxel treatment to possibly reduce the likelihood of the development of drug resistance.

## Abbreviations

SKOV3 and A2780: Taxol sensitive cell lines; TJ2500 and TA2780: Taxol resistant cell lines; RI: Resistant index; IC50: Half-inhibition concentration; HG-DMEM: HIGH-glucose medium; PI: Propidium iodide.

## Competing interests

The authors declare that they have no competing interests.

## Authors’ contributions

XQ Wang participated in the design of the study and drafted the manuscript. LY Pan conceived of the study and participated in its design and performed the statistical analyses. N Mao participated in the experimental design and implementation. LF Sun and XJ Qin participated in its design and coordination and performed the statistical analyses. J Yin helped to draft the manuscript. All authors read and approved the final manuscript.

## Authors’ information

XQ Wang worked in the Department of Obstetrics and Gynecology of Beijing Jishuitan Hospital as an attending physician and graduated from Peking Union Medical College Hospital with doctoral degree in 2008.

LY Pan worked in the Department of Obstetrics and Gynecology of Peking Union Medical College Hospital, Chinese Academy of Medical Sciences and Peking Union Medical College as a doctoral tutor and medical director.

N Mao worked in the Department of Cell Biology, Institute of Basic Medical Sciences, Academy of Military Medical Sciences as a director.

LF Sun worked in the Department of Obstetrics and Gynecology in Beijing Jishuitan Hospital as a medical director.

XJ Qin worked in the Department of Obstetrics and Gynecology in Beijing Jishuitan Hospital as vice-medical director.

J Yin worked in Department of Obstetrics and Gynecology of Peking Union Medical College Hospital as a physician.
